# Validation and Reliability of a Novel Test of Upper Body Isometric Strength

**DOI:** 10.1515/hukin-2015-0074

**Published:** 2015-10-14

**Authors:** David Bellar, Lena Marcus, Lawrence W. Judge

**Affiliations:** 1 School of Kinesiology, University of Louisiana at Lafayette, Lafayette LA.; 2 School of Physical Education, Sport and Exercise Science, Ball State University, Muncie IN.

**Keywords:** testing, measurement, force, bench press

## Abstract

The purpose of the present investigation was to examine the association of a novel test of upper body isometric strength against a 1RM bench press measurement. Forty college age adults (n = 20 female, n = 20 male; age 22.8 ± 2.8 years; body height 171.6 ± 10.8 cm; body mass 73.5 ± 16.3 kg; body fat 23.1 ± 5.4%) volunteered for the present investigation. The participants reported to the lab on three occasions. The first visit included anthropometric measurements and familiarization with both the upper body isometric test and bench press exercise. The final visits were conducted in a randomized order, with one being a 1RM assessment on the bench press and the other consisting of three trials of the upper body isometric assessment. For the isometric test, participants were positioned in a “push-up” style position while tethered (stainless steel chain) to a load cell (high frequency) anchored to the ground. The peak isometric force was consistent across all three trials (ICC = 0.98) suggesting good reliability. Multiple regression analysis was completed with the predictors: peak isometric force, gender, against the outcome variable 1RM bench press. The analysis resulted in a significant model (r2 = 0.861, p≤0.001) with all predictor variables attaining significance in the model (p<0.05). Isometric peak strength had the greatest effect on the model (Beta = 5.19, p≤0.001). Results from this study suggest that the described isometric upper body strength assessment is likely a valid and reliable tool to determine strength. Further research is warranted to gather a larger pool of data in regard to this assessment.

## Introduction

Learning is an essential part of the practice of a strength and conditioning professional (Petersen et al., 2011). In particular, testing that can determine information about strength is vital to the strength and conditioning professional and can assist in practice. Most speed and power sports place a premium on strength levels, which again places an emphasis on the need to be able to scientifically evaluate these characteristics (Haff, 2012; Judge et al., 2010; Judge et al., 2011; Kraska et al., 2009). It is therefore necessary to examine methodologies to test for strength (Newton and Dugan, 2002).

According to Newton and Dugan (2002) maximum strength is the testing of maximum force production and can be tested isometrically. It has been demonstrated that good association exists between isometric muscle actions and dynamic movements provided the joint angles are similar (Haff et al., 1997). Some isometric assessments have been shown to relate to on field performance in American football (Thompson et al., 2013). Additionally, Arslan (2005) demonstrated a relationship between power measures taken during a Wingate anaerobic test and isometric leg strength. There are a number of well-validated and reliable tests for maximum strength that examine isometric contractions (Bemben et al., 1992; Blazevich et al., 2002; Haff et al., 2005). The isometric mid-thigh pull has been well characterized in the literature (McGuigan et al., 2006). Briefly, the test consists of a subject who is fixed to a bar that is attached to a cage mounted over the top of a force platform. The subject is instructed to contract isometrically as hard and fast as possible and the force plate captures the data for subsequent analysis. Haff et al. (2005) examined this test in a group of elite female Olympic weightlifters. The results of this study demonstrated strong associations (r = 0.69 – 0.80) with actual Olympic lifting performance in the snatch, clean and jerk and competition total. These data collectively demonstrate a role for isometric assessment of strength through well-controlled tests.

However, most of the research to date has focused on strength testing in the lower body. There is little information about isometric testing for muscle in the upper body. Though the muscles of the lower limbs are of primary importance for sport performance, some athletes have a need for high levels of upper body strength such as shot putters (Judge and Bellar, 2012). For those researching the shot put or similar events where upper body strength is at a premium, the measurements are generally confined to free-weight tests of strength, which have precision limits based upon the adjustability of the weight set. The purpose of this study was to evaluate the reliability and validity of an isometric test of upper body strength using an instrument with much higher potential precision (load cell). Based upon the similar joint angles between the isometric test and the bench press, it was hypothesized that the results of a one-repetition maximum test would provide similar information at that garnered through the isometric assessment.

## Material and Methods

### Procedures

This investigation was conducted to determine if upper body isometric strength assessed via a load cell in a standardized body position was both a valid predictor of absolute strength and a reliable test. After a familiarization session where bench press technique was reviewed and practiced and the upper body isometric test (UBIST) was conducted, the participants were required to report to the lab for two testing days. The testing days were performed in a random order, and included a 1- repetition maximum bench press (1RM) assessment on one day and on another day measuring peak isometric force with the UBIST. All participants gave written informed consent to take part in the study and the methods were reviewed and approved by the Institutional Review Board at the University of Louisiana at Lafayette.

### Participants

Forty college age adults (n = 20 female, n = 20 male; mean ± SD; age 22.8 ± 2.8 years; body height 171.6 ± 10.8 cm; body mass 73.5 ± 16.3 kg; body fat 23.1 ± 5.4%) volunteered for the present investigation. The average 1RM on the bench press for the female participants was 45.9 ± 19.9 kg and 103.0 ± 16.7 kg for the males. Upon arrival at the lab, body height and mass were measured using a physicians triple beam balance and a stadiometer. A 3-site skinfold assessment was performed to determine body fat percentage. Additionally, a contractors t-square and slide were used to measure biacromial width, which was then used to standardize grip width and hand placement for the remainder of the lab visits.

### Upper Body Isometric Test (UBIST)

The participants were positioned on three elevated platforms with the chest directly suspended over a load cell anchored into the concrete floor of the lab (iLoad Pro, Loadstar Sensors, Fremont CA). The load cell chosen had a capacity of greater than 5000 newtons and a listed accuracy of 0.25% for the full scale of measurement. The participants were placed in a push-up style position, with the hands at 150% of biacromial width, and the elbows at 90 degrees of extension (measured via a goniometer). A thick, non-elastic strap was run over one shoulder and under the opposite shoulder and connected with metal rings to a chain that was tethered to the load cell. The participants were positioned so that no slack was apparent in the chain prior to initiation of data capture.

The participants were instructed to keep their backs flat, and push with their hands maximally until told to stop by the researcher. Prior to data capture, the load cell was tared to ensure the weight of the load cell and apparatus were accounted for. The researcher started data collection and verbally instructed the participant to “push as hard as possible”. The participants were verbally encouraged during data collection, which was terminated when the force production declined by 50 Newtons from the peak value registered (around 3 s in duration). The load cell was set to capture data at maximum rate (150 Hz) and the data was exported and analyzed in JMP 11.0 (SAS Institute Inc, Cary NC). Peak force values were isolated from the data and used for subsequent analysis. The test was performed three times with 5 min rest between assessments.

### One Repetition Maximum Assessment (1RM)

All bench press movements were performed at 150% of biacromial width (to match the hand position for UBIST) on the same equipment using a ParaBody bench (Brunswick Corporation, Lake Forest IL), a Rogue Fitness R4 power rack (Rogue Fitness, Columbus OH) and Ivanko Bar (Ivanko Barbell Company, San Pedro CA) and standard Olympic weight plates. Fractional plates (Piedmont Design Associates, Mooreville NC) were included to reduce the minimal size of adjustment in resistance to 0.11 kg as this has been demonstrated to enhance test performance (Bellar et al., 2013). During the familiarization session the participants bench press technique was assessed by an experienced strength and conditioning practitioner based upon the guidelines of the National Strength and Conditioning Association (NSCA). The assessment included having five points of contact (2 feet on the floor, and hips, shoulder and head on the bench surface). The participants were instructed to keep their hips on the bench and to lightly touch the bar to the chest prior to pressing the bar upward until a full range of motion was achieved. Based upon the familiarization session a weight was selected for each participant for an initial warm-up set of 8 repetitions. A second warm-up set of 8 repetitions was performed, followed by a set of 3 repetitions at increased resistance leading up to an estimated 80% effort. Afterwards 4 single attempts were used to determine the one repetition maximum. A rest time of 3 min was mandated between sets.

### Statistical Analysis

Data were analyzed for normality using Shapiro-Wilk tests. Repeated measures ANOVA and Intra-Class Correlations with typical error were used to assess reliability during the isometric upper body test sessions. Multiple regression analysis was used to examine the relationship between the one repetition bench press assessment and the upper body isometric test. All data analysis was performed using JMP 11.0 (SAS Institute Inc., Cary NC) or the Analysis of Reliability with a Spreadsheet (Hopkins, 2000).

## Results

### Analysis of Normality

Analysis of data using Shapiro-Wilks tests did not result in any data that deviated from normal distribution (W>0.969, p>0.342)

### Reliability of UBIST

Repeated measures ANOVA did not reveal a significant main effect of time (trial 1, 2, 3) during the upper body isometric strength test (F_1,37_ = 1.39, p=0.262). The Intra-Class Correlation coefficient similarly suggested that the test was a reliable measure (ICC = 0.98). Typical error was calculated at 0.11 meeting the criteria for small groups (0.2) according to Smith and Hopkins (2011). Based upon these data it appears the test has good reliability within day.

### Association of the UBIST with 1RM Bench Press

Multiple linear regression with the predictors peak force from the UBIST and sex were used to predict 1RM bench press strength. This resulted in a significant model (r^2^ = 0.861, p≤0.001) with both predictors being significant in the model (UBIST Beta = 0.519, p≤0.001; Sex Beta = 0.503, p≤0.001, RMSE = 12.76). When genders were analyzed separately, the UBIST for females was significantly associated with 1RM bench press (r^2^ = 0.422, p = 0.0019) and (r^2^ = 0.691, p ≤ 0.001) for males. The predicted values based upon the regression analysis were 52.4 ± 18.7 kg for females and 93.6 ± 21.7 kg for males using the regression equation:

1RM=17.7401289138412+0.11897785349582*UBIST peak.

## Discussion

In the world of sport performance a premium is placed on the measurement of strength characteristics in the lower body for obvious reasons. However, some sports require not only lower body strength, but also considerable upper body strength such as the shot put (Judge and Bellar, 2012). For these and similar, athletes tests of upper body strength that are reliable, valid and precise are warranted. It was due to the lack of these types of a test that the current study was undertaken.

The present investigation showed that upper body isometric assessment in a “push-up” style position using a load cell is a reliable and valid assessment of upper body strength. The intra class correlations and typical error suggest that the test is repeatable. Based upon the results it is likely to perform the test in duplicate, as the values were consistent across all three trials with the 5 min rest periods.

The UBIST was also strongly associated with the absolute strength measure performed. Though there is little available data in the literature in regard to upper body isometric testing, previous work by McGuigan et al. (2006) demonstrated similar correlations between an isometric mid-thigh pull (lower body) and 1RM measures (r values range: 0.73–0.97). This finding was similar to the reports of Beckham et al. (2013) who reported strong correlations between absolute peak force on an isometric mid-thigh pull and lifting performance for the Olympic lifts. While the present test mirrored these strong correlations with lifting performance, the results should be interpreted with caution. The difference in the predicted values from the regression analysis and the actual 1RM values suggested that the UBIST predicted 1RM strength with an average error of around 9.4 kg for men and 6.5 kg for women. While the test alone is likely a good test to determine upper body strength, it may not be a useful assessment to predict 1RM strength values in the bench press exercise. However, the UBIST has obvious advantages as a tool for research. The load cell can resolve differences on the order of 0.01 Newtons, which is far greater precision than tests using free-weights. This alone makes this test valuable to the sport science researcher who is interested in upper body strength development.

Though the findings in regard to the UBIST are promising, there are a number of areas that require further evaluation for this measure. Load cells generally sample at a rate that is slower than those normally encountered on force plate systems. Kraska et al. (2009) found difference in performance characteristics among stronger and weaker athletes, using isometric testing captured at 1000 Hz on a force plate. The present system used for UBIST samples at a modest 150 Hz rate. In the future, higher frequency systems should be evaluated as new technology becomes available. Higher frequency systems could result in data capture that could reduce some of the RMSE seen in the prediction of the 1RM bench press. Although, some of that error is likely do to small variations in technique employed in the bench press movement, as the body position was standardized in the UBIST.

It is generally accepted that speed and power athletes possess higher strength levels than normal for the general population. Even within speed and power athletes, higher strength levels are predictive of greater success (Judge and Bellar, 2012). The present work with the UBIST included normal college-age students, thus, further work will need to be conducted to evaluate the utility of this test for athletic populations.

Overall, the UBIST was shown to have promise as a reliable and valid alternative measure of upper body strength. Future research is warranted on larger population, and especially athletic populations. Additionally, as more advanced technology in load cells becomes available and affordable, higher sampling rates should be evaluated.

In conclusion, the present investigation suggests that the UBIST may be a useful test for the determination of upper body strength, particularly in laboratory testing. The materials necessary to conduct the test (load cell, platforms, harness) are not cost prohibitive and the test only takes a few minutes to administer. The test demonstrated good reliability and also has resolution down to less than 0.1 Newton, far greater precision than conventional strength tests using free weights. Provided that the subject is positioned as outlined above, the strength and conditioning professional or researcher could use this test to quickly assess upper body strength in a large group of subjects.

## Figures and Tables

**Figure 1:**
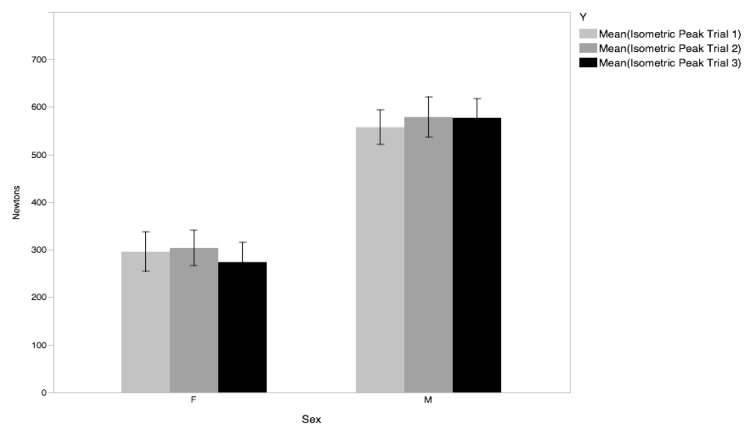
Chart of Peak Force on the UBIST by trial (1,2,3) by gender. Error bars represent ± 1 SE

**Figure 2:**
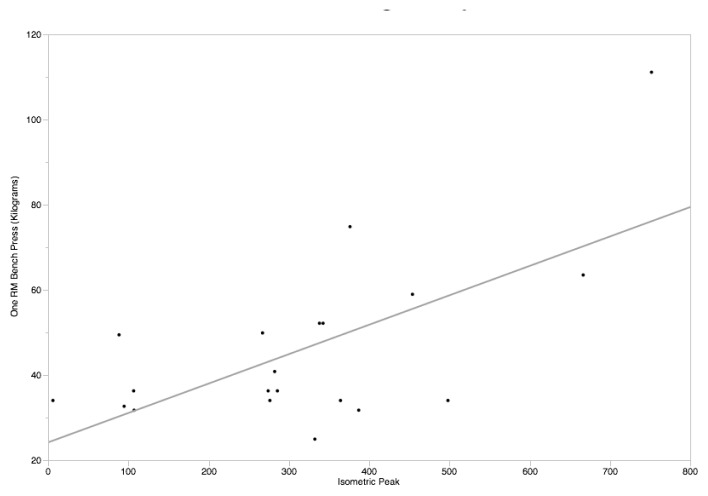
Scatterplot of Peak Force on the UBIST by 1RM Bench Press for female participants.

**Figure 3:**
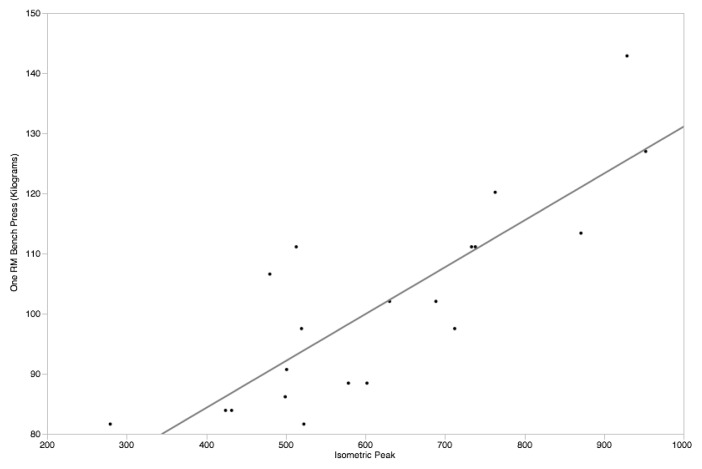
Scatterplot of Peak Force on the UBIST by 1RM Bench Press for male participants.
